# Ultrasound-guided Emergency Pericardiocentesis Simulation on Human Cadavers: A Scoping Review

**DOI:** 10.5811/westjem.39696

**Published:** 2025-05-19

**Authors:** Luca Ünlü, Felix Margenfeld, Adib Zendehdel, Johannes A. Griese, Amélie Poilliot, Magdalena Müller-Gerbl, Christian H. Nickel, Mirza Dedic

**Affiliations:** * University Hospital Basel, Department of Emergency Medicine, Petersgraben 2, CH-4031, Basel, Switzerland; † University of Basel, Department of Anatomy, Pestalozzistrasse 20, CH-4056, Basel, Switzerland

## Abstract

**Objectives:**

Emergency pericardiocentesis is a critical but infrequently performed procedure in emergency medicine, necessitating effective training modalities for emergency physicians. In this scoping review we aimed to identify existing literature on simulation of ultrasound-guided pericardiocentesis in human cadavers.

**Methods:**

We carried out a scoping review based on a search on the use of sonography on human cadavers. The following databases were searched: MEDLINE; EMBASE; CENTRAL; BIOSIS Previews; and Web of Science Core Collection. Additionally, we performed a gray literature search. Title and abstract screening were done by a single reviewer, and full-text review was performed by two independent reviewers. Studies included were limited to those published in English or German, focusing specifically on ultrasound-guided pericardiocentesis training models in human cadavers, with no restrictions on publication year or outcomes.

**Results:**

Our search strategy yielded 9,821 publications and 1,440 reports were assessed for eligibility. Ultimately, four studies met the inclusion criteria. All were conducted in the USA; two used soft-embalmed cadavers, one reported using fresh frozen cadavers, and one did not specify the cadaver type used. All studies accessed the pericardial sac using large-bore catheters or peripheral lines, filling it with (colored) water for simulation.

**Conclusions:**

Evidence on ultrasound-guided emergency pericardiocentesis simulation on human cadavers remains limited, but based on the four studies we reviewed human cadavers could be used for (emergency) pericardiocentesis simulation.

## INTRODUCTION

Emergency pericardiocentesis is a core emergency medicine (EM) skill covered in EM curricula such as the Emergency Medicine Key Index Procedure Minimums, the European Core Curriculum for Emergency Medicine and the 2022 Curriculum for the Fellowship of the Australasian College for Emergency Medicine.^1^–^3^ It is an infrequent, high-acuity-low-occurrence (HALO) procedure, and emergency physicians are expected to perform this skill in a resuscitative setting.^4^ Hence, it is essential that EM residency programs provide realistic training opportunities that facilitate learning and prepare emergency physicians to perform emergency pericardiocentesis. Currently, training is typically performed by using commercial or self-made pericardiocentesis simulators.^1^ However, simulators often lack realism in terms of haptic feedback and possible pericardiocentesis sites.

Previous research has demonstrated the superiority of cadaver training to simulator training for skills acquisition in other EM procedures, such as emergency front of neck access (eFONA) and tube thoracostomy.^5^ During the implementation of cadaveric emergency pericardiocentesis simulation at our own institution, we identified several challenges such as rapid leakage of fluid from the pericardial sac into the chest, breakdown of the simulation after a few punctures, and the quality of the pericardial effusion on ultrasound. To learn from the experience of others, we conducted a scoping review to systematically map the current body of literature. Our aim in this scoping review was to identify existing literature on simulation (concept) of ultrasound-guided pericardiocentesis (context) in human cadavers (population). The primary research question was: What is the existing literature regarding the use of human cadavers for simulation of ultrasound-guided (emergency) pericardiocentesis?

## METHODS

### Study Design

A protocol for this scoping review was registered in Open Science Framework Registries (osf.io/qby92) on September 27, 2024. This scoping review is based on a search on the use of sonography on human cadavers and was conducted in accordance with the Preferred Reporting Items for Systematic Reviews and Meta-Analyses extension for Scoping Reviews (PRISMA-ScR) guidelines.^6^

### Eligibility Criteria

This scoping review includes literature on ultrasound-guided pericardiocentesis simulation on human cadavers. The following types of publications were considered for inclusion: peer-reviewed articles; conference abstracts; dissertations; and textbook chapters. Only studies published in English or German were included, with no restrictions on publication year or study outcomes. We excluded articles focusing on animal studies, phantoms, or landmark-guided emergency pericardiocentesis. Additionally, we excluded all studies involving organ transplantation, as preliminary searches yielded many unrelated results in this area.

### Search Strategy

We developed a search strategy in consultation with a data librarian to generate a database on the use of sonography on human cadavers. (See Table 1 in the online appendix.) The search process for this database was conducted on January 3, 2023. The results were updated by a search performed on June 20, 2024. The following databases were searched: MEDLINE, EMBASE, CENTRAL, BIOSIS Previews, and Web of Science Core Collection. Additionally, gray literature was searched in the National Grey Literature Collection. Furthermore, dissertations and PhD theses were retrieved from the Electronic Theses Online Service (EThOS) and the Open Access Theses and Dissertations (OATD) database. We reviewed the reference lists of all studies meeting the inclusion criteria for this scoping review for additional relevant publications.

**Population Health Research Capsule**

What do we already know about this issue?

*Emergency pericardiocentesis is a core emergency medicine skill, but because it is infrequently performed realistic training opportunities are needed.*

What was the research question?

*What is the existing literature regarding the use of human cadavers for simulation of ultrasound-guided emergency pericardiocentesis?*

What was the major finding of the study?

*In four studies, human cadavers were used for ultrasound-guided emergency pericardiocentesis simulation.*

How does this improve population health?

*Cadaver-based simulation of emergency)pericardiocentesis seems feasible and could improve procedural competency.*

### Study Selection

Following the search, we collated all identified citations using Endnote X9 (Clarivate, Philadelphia, PA).^7^ Duplicates were removed by Endnote following Bramer and Giustini^8^ and then manually removed when further duplicates were found later in the review process. In the process of developing a cadaver lab for sonography on human cadavers, we wanted to learn from the experience of others. For this reason, we used a broad search strategy to generate a database on the use of sonography on human cadavers. We performed title and abstract screening in Endnote X9. The initial title and abstract screening were conducted by a single reviewer, who evaluated the search results against the minimum inclusion criteria of the database on the use of sonography on human cadavers. In the event of uncertainty, a second reviewer was consulted. To determine eligibility, two independent reviewers performed full-text review. To make the generated database on sonography on human cadavers more user-friendly, we ordered all citations according to the organ structures that the identified study was written on. For this purpose, one reviewer generated a list of 12 main categories and 225 sub-categories of anatomical structures (Table 2 in the online appendix) and ordered each study into the corresponding folder. For this scoping review, all studies from the “cardiac” folder were reviewed in full text by two independent reviewers against the inclusion and exclusion criteria. Disagreements were resolved by involvement of a third reviewer at each stage.

### Data Extraction and Synthesis

The following data were extracted from each article when available: authors; year of publication; journal in which the article was published; type of publication; country of origin; simulation setting; and evaluation of the simulation. Two extractors independently extracted the above-mentioned items, and their data were synthesized into a single table that summarized key findings. Additionally, a narrative review of the results of each included study was performed. In cases of disagreement, this was resolved through discussion.

## RESULTS

### Study Selection

**Figure 1:**
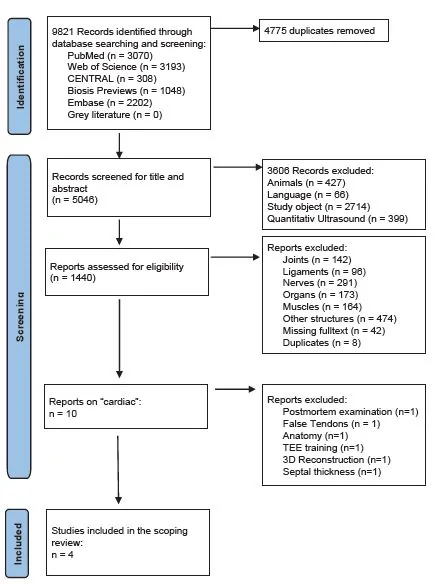
PRISMA flowchart. *PRISMA-ScR*, Preferred Reporting Items for Systematic Reviews and Meta-Analyses extension for Scoping Reviews; *CENTRAL*, Cochrane Central Registration of Trials; *TEE*, transesophageal echogardiography.

The search yielded a total of 9,821 results, of which 4,775 were identified as duplicates and subsequently removed. A total of 1,440 records were subjected to full-text screening for eligibility. Of these, 10 publications were categorized into the “cardiac” folder, of which four publications were deemed eligible for inclusion. (See PRISMA flowchart in Figure 1.)

### Study Characteristics of Included Articles

All four included studies were written in English and were conducted in the USA. Two of the studies were published as conference abstracts, while the other two were published as original research articles. All studies were published in peer-reviewed journals, with two published in 2012, one in 2013, and one in 2023. None of the four studies indicated sources of funding. Three of the four studies were conducted by emergency physicians, while the remaining study was conducted by cardiologists. A comprehensive overview of the studies, including methodologies and key findings, is presented in Table 1.

### Access to the Pericardial Sac with a Large-bore Catheter

Two studies report on the use of a large-bore catheter such as a sheath or a paracentesis catheter to fill the pericardial sac with fluid to simulate pericardial effusion.^9^,^10^ Fenstad et al used fluoroscopy to insert a guidewire through the left subclavian vein into the right ventricle (RV) where the RV free wall was pierced to access the pericardial space. Subsequently, a sheath was advanced into the pericardial space and water (100–500 milliliters) was injected into the pericardial sac. In another study, a right-sided thoracotomy was performed, and a large-bore paracentesis catheter was inserted into the pericardial sac.^10^ For this purpose a number 11 scalpel was used to create a nick in the pericardial sac and the large-bore paracentesis catheter was sutured to the pericardial sac. In this study, a normal saline bag hanging against gravity was used to fill the pericardial sac.

### Access to the Pericardial Sac with a Peripheral Venous Cannula

One study reported on the use of a peripheral venous cannula to fill the pericardial sac with fluid.^11^ In that study, a left-sided 3–4 centimeter mini thoracotomy was performed in the 5th intercostal space just over the cardiac apex. The left lung was retracted until the pericardial sac and phrenic nerve were identified. Using toothless forceps, an 18-gauge peripheral venous catheter was inserted into the pericardial sac. The peripheral venous catheter was sutured to the intercostal soft tissues and connected to a bag of normal saline, which was infused with a pressure bag.

### Filling of the Pericardial Sac

Three of the four studies specified that they used an intravenous (IV) bag to fill the pericardial sac with fluid.^10^–^12^ Inboriboon et al dyed the IV fluid with methylene blue. This allowed differentiation between a pericardial puncture (blue) and a cardiac puncture (red).^10^ One study specified that they used an IV-pressure bag.^11^

### Cadaver Type

In two studies soft cadavers were used; these cadavers were preserved by soft embalming rather than through a formalin-based fixation.^10^,^11^ In contrast to traditional formaldehyde embalmment cadavers, soft-embalmed cadavers retain tissue flexibility and texture, which makes them especially suitable for procedural training. Another study used fresh-frozen cadavers.^12^ One study did not further specify what cadaver type they used.^9^

### Efficacy of Simulation and Quality of Pericardial Effusion on Ultrasound Image

Two manuscripts additionally reported on the efficacy of their simulation. In one study, participants were asked to rate their overall course satisfaction on a 5-point Likert scale (5 is very satisfied, 1 is very dissatisfied). Likert-scale ratings were completed by 28 of 36 participants (78%), and the overall course satisfaction was rated to be 4.94.^9^ The second manuscripts noted that all nine participants were able to visualize the pericardiocentesis needle under dynamic ultrasound guidance and that the model did not demonstrate evidence of breakdown after approximately 40–50 punctures. The other manuscripts did not provide information on the quality of the pericardial effusion on ultrasound image. Moreover, the participants were asked to rate their pre- and post-training procedural confidence on a Likert scale from 0–7, where 7 is best. The average pre-training procedural confidence was 2.1 + 0.9, the average post-training procedural confidence 5.3 + 0.6 with an absolute change of +3.2 (95% CI 2.2–4.3, *P* <0.001). The participants were also asked to provide qualitative feedback. Here, the participants highlighted realism of the training model. However, only one of nine participants had previously performed an (emergency) pericardiocentesis in real life.^11^

## DISCUSSION

This scoping review identified four studies that report on the use of a cadaver model for ultrasound-guided emergency pericardiocentesis simulation. Our scoping review indicates that both soft and fresh-frozen cadavers can be used for cadaver-based emergency pericardiocentesis simulation. Access to the pericardium was achieved using either large-bore catheters or peripheral venous catheters, with fluid infusion facilitated by IV bags hung against gravity or by using a pressure bag.

Different types of human cadavers are used in medical education, each offering distinct advantages and disadvantages. Formalin-embalmed cadavers are ideal for long-term preservation, but the stiffness induced by formalin compromises their natural flexibility.^13^ In contrast, soft-embalmed cadavers maintain a more natural texture. However, they are susceptible to mold if not stored correctly, and their embalming process is more complex and costly compared to formalin-embalmed cadavers.^14^ Soft-embalmed cadavers were rated to provide a higher degree of realism than formalin-embalmed cadavers in a study on cadaver-based thyroid surgery training.^15^ Another option is a fresh-frozen cadaver. They are preserved shortly after death without chemical embalming and retain the texture, color, and structural integrity of fresh tissues. However, their handling and storage present challenges, and they must be thawed prior to use.^13^ Often, cadavers are from older people, who typically experience sarcopenia, a natural age-related loss of muscle mass. In contrast, patients of many resuscitative procedures such as resuscitative thoracotomy are predominantly young and male.^17^ Hence, some resuscitative procedures (such as resuscitative thoracotomy) are less physically demanding in cadaver labs than in real life.^18^ However, for emergency pericardiocentesis training on human cadavers, this is usually not an issue, as the haptic feedback during the procedure closely resembles that of living patients. Access to cadavers remains a barrier as most cadaver labs located at universities, compounded by limited supply and the high costs associated with preparation, storage, and eventual burial.^19^ Nonetheless, there are numerous commercial and academic, human cadaver-based courses for resuscitative procedures such as resuscitative thoracotomy. However, human cadavers seem not yet to be widely used for emergency pericardiocentesis simulation.^18^

Participants of emergency pericardiocentesis simulations on human cadavers rated themselves to be more confident in the procedure and highlighted the realism of the simulation on human cadavers.^11^ While a positive self-evaluation does not necessarily translate to proficiency in performing resuscitative interventions, previous research indicates that physicians are less likely to commence resuscitative procedures when they lack confidence in performing them, highlighting that perceived self-efficacy is a determinant of performance in resuscitative practice.^20^ Procedural competency for other life-saving procedures such as rapid sequence intubation (RSI) or tube thoracostomy can be acquired by training in the emergency department, as these are required sufficiently frequently.^21^ However, emergency pericardiocentesis is a rare procedure that might even have to be performed without the back-up of a colleague with prior experience in emergency pericardiocentesis.

### Learning Points from this Scoping Review for Our Own Cadaver Lab

This scoping review provided valuable insights for developing a soft-embalmed cadaver emergency pericardiocentesis simulation. Previous simulations at our institution faced issues such as fluid leakage from the pericardial sac, simulation breakdown after a few punctures, and poor ultrasound quality of the pericardial effusion.

**Figure 2:**
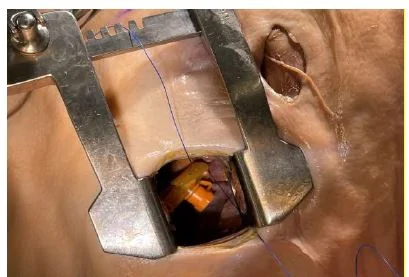
Right-sided mini-thoracotomy in the 4th intercostal space at the anterior axillary line. A 14G peripheral venous cannula is sutured into the pericardium, as described in Table 2.

**Figure 3:**
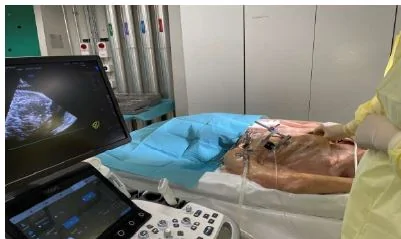
Simulation setup. The peripheral venous catheter in the pericardial sac and the subclavian central line are both connected to a pressure infusion via IV tubing.

Building upon this scoping review and prior experience, a new simulation was developed. All consent requirements were met in accordance with Article 36 of the Human Research Act (HRA), and prior determination of death was conducted as per Article 37, paragraph 1 HRA. In this simulation, the pericardial sac is continuously filled with fluid through a pressured infusion via a right-sided mini-thoracotomy (see Figures 2 and 3). As in previous experiments the heart appeared as a single mass on ultrasound, the heart chambers were filled with fluid to enable their identification. Additionally, the left hemithorax was filled with fluid to facilitate pericardiocentesis training from all three approaches (subxiphoidal, apical, and parasternal). The abdomen in soft-embalmed cadavers is usually collapsed, which potentially makes subxiphoid pericardiocentesis easier than in real patients. To mitigate this, fluid was introduced into the abdominal cavity to create a more distended appearance. Detailed setup instructions are provided in Table 2.

The simulation setup yielded ultrasound images consistently rated as highly realistic by all authors with real-world experience in emergency pericardiocentesis. Notably, even after approximately 50 punctures and dilations, no structural breakdown was observed in the cadaver model. Importantly, the modifications to the soft-embalmed cadaver did not compromise the realism of training for other resuscitative procedures. This was crucial, as both ethical and financial considerations must be carefully weighed when using human cadavers. The right-sided mini-thoracotomy used to fill the pericardial sac did not interfere with resuscitative thoracotomy training. In fact, the presence of fluid in the left chest and pericardial sac enhanced the fidelity by simulating pathologies commonly encountered during resuscitative thoracotomy for traumatic cardiac arrest (TCA). Additionally, the cadaver allowed for the identification of TCA-associated pathologies with ultrasound, such as free fluid in the pericardium, left chest, and abdomen.

Further research should evaluate the quality of the effusion as observed on ultrasound, as well as participants’ perceived procedural competency following the simulation. Moreover, authors should focus on providing a well-documented methodology for their human cadaver-simulation setup. By these means, other programs can replicate the setup and benefit from the insights gained.

## LIMITATIONS

This scoping review was conducted based on a database of ultrasound-guided procedures on human cadavers. Hence, a very broad search strategy was used to generate this database. (See Table 1 in the online appendix.) During the initial screening process, many irrelevant citations related to transplantation were found, prompting the addition of the Boolean operator “NOT transplantation” to the search string. While this might have excluded studies that involved the transplantation of other tissues into human cadavers for emergency pericardiocentesis training, it seems unlikely that this substantially impacted our results.

## CONCLUSION

Evidence on ultrasound-guided emergency pericardiocentesis simulation on cadavers remains limited. However, four studies show that it is feasible to use human cadavers for simulation of (emergency) pericardiocentesis.

## Supplementary Information



## Figures and Tables

**Table 1:** Characteristics of included studies.

Authors	Year	Journal	Publication type	Country of origin	Simulation Set-up	Evaluation of the simulation
Fenstad E et al^9^	2012	*Critical Care Medicine*	Conference (Abstract)	USA	Cadaver (not further specified), fluoroscopy guided puncture of the RV-free wall trough the left subclavian vein. Insertion of a sheath into the pericardial sac, injection of variable amounts of water (100–500 ml).	Likert-scale ratings, where 5 indicates very satisfied and 1 indicates very dissatisfied, were completed by 28 out of 36 participants (78%). The overall course satisfaction rating was 4.94.
Friedman TA et al^12^	2013	*Annals of Emergency Medicine*	Conference (Abstract)	USA	Fresh frozen cadavers were used, with a catheter placed in the pericardial sac and a hanging bag of saline. Methods were not further specified.	No evaluation of the simulation.
Inboriboon, PC et al^10^	2012	*Journal of Emergency Medicine*	Research Article (“How to”)	USA	Soft cadavers were used, with a right-sided thoracotomy performed to introduce a large-bore paracentesis catheter into the pericardial sac. A saline bag was hung to gravity and attached to the catheter.	No evaluation of the simulation.
Oskar K et al^11^	2023	*Journal of Education & Teaching in Emergency Medicine*	Research Article (“How to”)	USA	Soft cadavers were used, with a left-sided apical mini-thoracotomy performed. An 18-gauge angiocath was introduced into the pericardium and sutured to the intercostal soft tissue. A saline bag was attached to the catheter and infused using a pressure bag.	All nine participants were able to visualize the needle under dynamic ultrasound guidance. The model demonstrated no evidence of breakdown after approximately 40 to 50 punctures.

*USA*, United States of America; RV, right ventricle.

**Table 2:** Materials and instructions for setting up human cadaver emergency pericardiocentesis simulation.

**Materials:** 1x DeBakey (mini-Finochietto) rib spreader 1x 14G peripheral venous cannula (Vasofix Safety, B. Braun Melsungen SE, Melsungen, Germany) 2x 10G peripheral venous cannula (BD angiocath Becton, Dickinson and Company, Franklin Lakes, NJ, USA) 4x IV-giving set (Intrafix SafeSet, B. Braun Melsungen SE, Melsungen, Germany) Approx. 9x 1000mL bag of normal saline 1x 3-way-stopcock with 10cm connection tubing (Discofix, B. Braun Melsungen SE, Melsungen, Germany) 4x 1000mL pressure infusion bag 1x red food dye 1x 8Fr two-lumen-central line (with Blue FlexTip, Arrow International, Inc, Reading, PA, USA) 2x 2.0 monofilament sutures (SERALON blau monofil, Serag-Wiessner, GmbH & Co, Naila, Germany) 1x DeBakey 25cm needle holder 1x DeBakey atraumatic dissecting forceps 1x Scalpel No 10
1. **Filling of the Pericardial Sac:** An approximately 6 cm incision is made at approximately the 4th right intercostal space at the anterior axillary line. The intercostal muscles are dissected from the rib until the right lung is exposed. Using a needle holder, the lung is gently pressed downward to expose the pericardium. A 14G peripheral venous cannula is then inserted into the pericardial sac and secured with sutures (Figure 2). A 3-way stopcock is attached so that the IV tubing can be more easily connected to the cannula, and 1–2L of normal saline is infused using a pressure bag. Red food dye may be added to enhance realism. The infusion must remain pressurized throughout the pericardiocentesis simulation.
2. **Filling of the Heart Chambers:** A right-sided subclavian central line is inserted using an open surgical technique. If large blood clots are present in the subclavian vein, they must be removed using forceps to allow central line insertion. A pressure bag is then attached to the central line to fill the heart chambers, allowing proper visualization of the heart and its chambers on ultrasound imaging.
3. **Filling of the Left Hemithorax:** A 10G peripheral venous cannula is placed in the 4th left intercostal space at the mid-axillary line. A pressure bag is used to infuse 2–3L of normal saline until the left hemithorax is completely filled. This step is essential for enabling parasternal and apical ultrasound imaging.
4. **Filling of the Abdominal Cavity:** A 10G peripheral venous cannula is inserted into the abdominal cavity. Approximately 3L of normal saline are infused via a pressure bag until the abdomen becomes distended.
