# Supplementary material for: Ultrasound-guided Emergency Pericardiocentesis Simulation on Human Cadavers: A Scoping Review
Source: West J Emerg Med. 2025 May 19;26(3):685–91. doi: 10.5811/westjem.39696 (PMC12208006; doi:10.5811/westjem.39696)
Supplement: Supplementary file 1 [file wjem-26-685-s001.docx]

**ONLINE APENDIX**

**Table 1.** PubMed Search strategy.

Strategies for other databases are provided in the study protocol registered with OSF Registry (osf.io/qby92).

| **PubMed:**  (ultrasonography[mesh] OR ultrasonography[tiab] OR ultraso*[tiab]) AND (cadaver[mesh] OR cadaver*[tiab] OR corpse*[tiab] OR dead bod*[tiab] OR donated bod*[tiab] OR body donation*[tiab] OR deceased*[tiab] OR lifeless[tiab]) NOT (animals[mesh] NOT humans[mesh]) NOT (animal*[tiab] NOT human*[tiab])  During scanning process too many irrelevant transplantation studies were found, so the search string had been narrowed down. (+ NOT transplantation)  (ultrasonography[mesh] OR ultrasonography[tiab] OR ultraso*[tiab]) AND (cadaver[mesh] OR cadaver*[tiab] OR corpse*[tiab] OR dead bod*[tiab] OR donated bod*[tiab] OR body donation*[tiab] OR deceased*[tiab] OR lifeless[tiab]) NOT (animals[mesh] NOT humans[mesh]) NOT (animal*[tiab] NOT human*[tiab]) NOT  (transplantation[mesh]) NOT (transplantation[tiab])  Results: 3070 (date: 20.06.2024) |
| --- |
| **MeSH Terms:**  (ultrasonography[mesh] OR ultrasonography[tiab] OR ultraso*[tiab]) AND (cadaver[mesh]  OR cadaver*[tiab] OR corpse*[tiab] OR dead bod*[tiab] OR donated bod*[tiab] OR body  donation*[tiab] OR deceased*[tiab] OR lifeless[tiab]) NOT (animals[mesh] NOT humans[mesh]) NOT (animal*[tiab] NOT human*[tiab]) |

**Table 2.** List of anatomical structures used for the database. The number of sub-categories for each main category is provided in brackets.

| - **Ear** - **Eye** - **Veins (5)**   - Femoral vein   - Saphenous vein   - Subclavian vein   - Jugular vein   - Tibial and fibular veins - **Ligaments and tendons (34)**   - Achilles tendon   - Dorsal hood of the hands   - Iliolumbar membrane   - Patellar ligament   - Anterolateral ligament of the knee   - Pulleys   - Calcaneofibular ligament   - Plantar calcaneonavicular ligament   - Transverse carpal ligament   - Lateral collateral ligament of the knee   - Radial collateral ligament of the elbow   - Medial collateral ligament of the knee   - Ulnar collateral ligament of the elbow   - Ulnar collateral ligament of the thumb   - Coracoacromial ligament   - Coracohumeral ligament   - Yellow ligament   - Glenohumeral ligament   - Intermetatarsal ligament   - Pisotriquetral ligament   - Popliteofibular ligament   - Posterior sacroiliac ligament   - Scapholunate ligament   - Anterior talofibular ligament   - Talonavicular ligament   - Proximal tibiofibular ligament   - Ulnar and radial collateral ligament of the wrist   - Dorsal and palmar carpal ligaments of the wrist   - Interosseous membrane of the forearm   - Extensor retinaculum of the wrist   - Patellar retinaculum   - Spring ligament   - Distal tibiofibular syndesmosis   - Iliotibial band - **Other (36)**   - Abdominal wall   - Abscess   - Anus   - Bone   - Bursa of medial collateral ligament of the knee   - Bursae   - Costal cartilage   - Denonvilliers space   - Diaphragm   - Pelvic diaphragm   - Douglas pouch   - Extraperitoneal spaces   - Fabella and cyamella   - Antebrachial fascia   - Crural fascia   - Gluteal fascia   - Iliac fascia   - Fat and soft tissue   - Feet   - Fetus   - Gruberi bursa   - Hallucal interphalangeal ossicle   - Hunter canal   - Lymph nodes   - Nose   - Pacinian corpuscles   - Parenchymal organs   - Peritoneal cavity   - Pleura   - Retroperitoneal space   - Scarpa fascia   - Several structures   - Soft palate   - Thorax   - Tongue and mouth   - Unclear - **Joints (23)**   - Acromioclavicular joint   - Atlantooccipital joint   - Carpometacarpal joint   - Costotransverse joint   - Hip joint   - Cubital joint   - Knee joint   - Glenoid joint   - Lisfranc joint   - Metacarpophalangeal joint   - Metatarsophalangeal joint   - Radiocarpal joint   - Distal radioulnar joint   - Sacroiliac joint   - Scaphotrapezial joint   - Sternoclavicular joint   - Subtalar joint   - Talocrural joint   - Talonavicular joint   - Craniomandibular joint   - Proximal tibiofibular joint   - Trapeziometacarpal joint   - Zygapophyseal joint - **Bones (16)**   - Calcaneus   - Clavicle   - Humerus   - Leg   - Mandible   - Maxilla   - Metatarsal   - Patella   - Pelvis   - Radius   - Rib   - Sacrum   - Several   - Spine   - Sternum   - Tibia - **Central Nervous System (11)**   - Brain   - Cerebral vessels   - Subarachnoid cisterns   - Accessory nerve   - Facial nerve   - Glossopharyngeal nerve   - Hypoglossal nerve   - Optic nerve   - Trigeminal nerve   - Vagus nerve   - Spinal canal - **Muscles (42)**   - Axillary arch muscle   - Hamstrings   - Adductor longus muscle   - Articular muscle of the knee   - Biceps brachii muscle   - Deltoid muscle   - Extensor carpi radialis longus muscle   - Extensor carpi radialis brevis muscle   - Extensor pollicis brevis muscle   - Extensor pollicis longus muscle   - Flexor digitorum muscle   - Gastrocnemius muscle   - Gluteus medius muscle   - Gracilis muscle   - Iliopsoas muscle   - First interosseus muscle of the hand   - Levator ani muscle   - Masseter muscle   - Mylohyoid muscle   - Obliquus externus abdominis muscle   - Obliquus capitis inferior muscle   - Obturator internus muscle   - Orbicularis oris muscle   - Palmaris brevis muscle   - Palmaris longus muscle   - Pectoralis major muscle   - Fibularis longus muscle   - Piriformis muscle   - Plantaris muscle   - Polpliteus muscle   - Pronator quadratus muscle   - Quadriceps femoris muscle   - Rectus abdominis muscle   - Sphincter ani externus muscle   - Sternocleidomastoid muscle   - Tibialis anterior muscle   - Tibialis posterior muscle   - Trapezius muscle   - Extensor digitorum muscle   - Platysma   - Rotator cuff muscles   - Several - **Nerves (38)**   1. C5 root   2. Dorsal root   3. Superior cervical ganglion   4. Celiac ganglia   5. Stellate ganglion   6. L5 root   7. Axillary nerve   8. Lateral antebrachial cutaneous nerve   9. Lateral femoral cutaneous nerve   10. Posterior femoral cutaneous nerve   11. Dorsal scapular nerve   12. Femoral nerve   13. Iliohypogastric and ilioinguinal nerves   14. Intercostal nerve   15. Posterior antebrachial interosseus nerve   16. Sciatic nerve   17. Median nerve   18. Obturator nerve   19. Greater occipital nerve   20. Lesser occipital nerve   21. Pectoral nerve   22. Phrenic nerve   23. Pudendal nerve   24. Radial nerve   25. Saphenous nerve   26. Subcostal nerve   27. Suprascapular nerve   28. Long thoracic nerve   29. Transverse cervical nerve   30. Ulnar nerve   31. Superior cluneal nerves   32. Cutaneous nerves of the hand   33. Brachial plexus   34. Celiac plexus   35. Hypogastric plexus   36. Lumbosacral plexus   37. Dorsal branches of the spinal nerve   38. Several - **Artery (11)**   - Carotid artery   - Middle cerebral artery   - Coronary artery   - Femoral artery   - Iliac artery   - Popliteal artery   - Pulmonal artery   - Renal artery   - Spinal artery   - Aorta   - Several arteries - **Organs (23)**   - Adrenal gland   - Breast   - Large intestine   - Esophagus   - Stomach   - Heart   - Kidney   - Larynx   - Liver   - Lung   - Pancreas   - Penis   - Prostate gland   - Salivary gland   - Several   - Spleen   - Testicles   - Thymus   - Thyroid gland   - Trachea   - Urogenital tract   - Uterus   - Vagina |
| --- |

Table 3. Preferred Reporting Items for Systematic reviews and Meta-Analyses extension for Scoping Reviews (PRISMA-ScR) checklist.

| **SECTION** | **ITEM** | **PRISMA-ScR CHECKLIST ITEM** | **REPORTED ON PAGE #** |
| --- | --- | --- | --- |
| **TITLE** | | | |
| Title | 1 | Identify the report as a scoping review. | Title page |
| **ABSTRACT** | | | |
| Structured summary | 2 | Provide a structured summary that includes (as applicable): background, objectives, eligibility criteria, sources of evidence, charting methods, results, and conclusions that relate to the review questions and objectives. | 2 |
| **INTRODUCTION** | | | |
| Rationale | 3 | Describe the rationale for the review in the context of what is already known. Explain why the review questions/objectives lend themselves to a scoping review approach. | 4 |
| Objectives | 4 | Provide an explicit statement of the questions and objectives being addressed with reference to their key elements (e.g., population or participants, concepts, and context) or other relevant key elements used to conceptualize the review questions and/or objectives. | 4 |
| **METHODS** | | | |
| Protocol and registration | 5 | Indicate whether a review protocol exists; state if and where it can be accessed (e.g., a Web address); and if available, provide registration information, including the registration number. | 5 |
| Eligibility criteria | 6 | Specify characteristics of the sources of evidence used as eligibility criteria (e.g., years considered, language, and publication status), and provide a rationale. | 5 |
| Information sources* | 7 | Describe all information sources in the search (e.g., databases with dates of coverage and contact with authors to identify additional sources), as well as the date the most recent search was executed. | 5/6 |
| Search | 8 | Present the full electronic search strategy for at least 1 database, including any limits used, such that it could be repeated. | Appendix tab.1 |
| Selection of sources of evidence† | 9 | State the process for selecting sources of evidence (i.e., screening and eligibility) included in the scoping review. | 6 |
| Data charting process‡ | 10 | Describe the methods of charting data from the included sources of evidence (e.g., calibrated forms or forms that have been tested by the team before their use, and whether data charting was done independently or in duplicate) and any processes for obtaining and confirming data from investigators. | 6/7 |
| Data items | 11 | List and define all variables for which data were sought and any assumptions and simplifications made. | 6/7 |
| Critical appraisal of individual sources of evidence§ | 12 | If done, provide a rationale for conducting a critical appraisal of included sources of evidence; describe the methods used and how this information was used in any data synthesis (if appropriate). | N/A |
| Synthesis of results | 13 | Describe the methods of handling and summarizing the data that were charted. | 7 |
| **RESULTS** | | | |
| Selection of sources of evidence | 14 | Give numbers of sources of evidence screened, assessed for eligibility, and included in the review, with reasons for exclusions at each stage, ideally using a flow diagram. | 8 |
| Characteristics of sources of evidence | 15 | For each source of evidence, present characteristics for which data were charted and provide the citations. | 9 |
| Critical appraisal within sources of evidence | 16 | If done, present data on critical appraisal of included sources of evidence (see item 12). | N/A |
| Results of individual sources of evidence | 17 | For each included source of evidence, present the relevant data that were charted that relate to the review questions and objectives. | 9-12 |
| Synthesis of results | 18 | Summarize and/or present the charting results as they relate to the review questions and objectives. | 10 (Tab.1) |
| **DISCUSSION** | | | |
| Summary of evidence | 19 | Summarize the main results (including an overview of concepts, themes, and types of evidence available), link to the review questions and objectives, and consider the relevance to key groups. | 13 |
| Limitations | 20 | Discuss the limitations of the scoping review process. | 15 |
| Conclusions | 21 | Provide a general interpretation of the results with respect to the review questions and objectives, as well as potential implications and/or next steps. | 16 |
| **FUNDING** | | | |
| Funding | 22 | Describe sources of funding for the included sources of evidence, as well as sources of funding for the scoping review. Describe the role of the funders of the scoping review. | 9, title page |

* Where *sources of evidence* (see second footnote) are compiled from, such as bibliographic databases, social media platforms, and Web sites.

† A more inclusive/heterogeneous term used to account for the different types of evidence or data sources (e.g., quantitative and/or qualitative research, expert opinion, and policy documents) that may be eligible in a scoping review as opposed to only studies. This is not to be confused with *information sources* (see first footnote).

‡ The frameworks by Arksey and O’Malley (6) and Levac and colleagues (7) and the JBI guidance (4, 5) refer to the process of data extraction in a scoping review as data charting*.*

§ The process of systematically examining research evidence to assess its validity, results, and relevance before using it to inform a decision. This term is used for items 12 and 19 instead of "risk of bias" (which is more applicable to systematic reviews of interventions) to include and acknowledge the various sources of evidence that may be used in a scoping review (e.g., quantitative and/or qualitative research, expert opinion, and policy document).

*JBI*, Joanna Briggs Institute; *PRISMA-ScR*, Preferred Reporting Items for Systematic reviews and Meta-Analyses extension for Scoping Reviews.

*From:* Tricco AC, Lillie E, Zarin W, O'Brien KK, Colquhoun H, Levac D, et al. PRISMA Extension for Scoping Reviews (PRISMAScR): Checklist and Explanation. *Ann Intern Med*. 2018;169:467–473. [doi: 10.7326/M18-0850](http://annals.org/aim/fullarticle/2700389/prisma-extension-scoping-reviews-prisma-scr-checklist-explanation)
